# Neural and Oxidative-Stress Parameters as Early Biomarkers of Hand–Arm Vibration Syndrome

**DOI:** 10.3390/biom16020238

**Published:** 2026-02-03

**Authors:** Zifei Tang, Qian Chen, Jia Li, Kanshou Zhou, Fanfei Zeng, Hongyu Yang

**Affiliations:** 1 School of Public Health, Guangdong Pharmaceutical University, Haizhu District, Guangzhou 510006, China; 2112415017@stu.gdpu.edu.cn (Z.T.); 2112415013@stu.gdpu.edu.cn (Q.C.); 2112515016@stu.gdpu.edu.cn (J.L.); 2112341034@stu.gdpu.edu.cn (K.Z.); 2112341023@stu.gdpu.edu.cn (F.Z.); 2 Guangdong Provincial Engineering Research Center of Public Health Detection and Assessment, Guangzhou 511400, China

**Keywords:** hand–arm vibration syndrome, vibration-induced white finger, vascular injury, oxidative stress, neurological indices

## Abstract

**Objective**: Investigate alterations in the expression of specific and sensitive biomarkers of oxidative stress (OS) in blood and in nerves during hand–arm vibration syndrome (HAVS) progression. **Methods**: Fifty workers with vibration-induced white finger (VWF) symptoms and exposure to hand vibration were selected from a Chinese factory by judgment sampling. Fifty workers not exposed to hand vibration served as a control group. Expression of OS-related indices in blood was measured. The same method was used to select 40 workers separately for the determination of neurological indicators. Blood samples were collected from participants, and expression of indicators in plasma was measured by ELISAs. **Results**: The receiver operating characteristic (ROC) curves of OS-related indices and neurological indices were analyzed to assess their diagnostic sensitivity to VWF. Among OS indices, the area under the ROC curve (AUC) of malondialdehyde (MDA), superoxide dismutase (SOD), glutathione (GSH), and glutathione peroxidase (GSH-PX) was >0.9. With regard to the neurological indices, the AUC of S100β, interleukin (IL)-10, creatine kinase (CK), and growth differentiation factor (GDF)-15 was 0.7–0.9. **Conclusions**: MDA, SOD, GSH, GSH-PX, S100β, IL-10, CK, and GDF-15 could be diagnostic markers for VWF.

## 1. Introduction

Hand–arm vibration syndrome (HAVS) is an occupational disorder. The typical symptoms of HAVS encompass paleness of the fingers, Raynaud’s phenomenon, as well as abnormal manifestations stemming from damaged nerve fibers and mechanical receptors [1]. The diagnosis of HAVS relies on meticulous evaluation of the paleness of fingers and electromyography [2]. The vascular element of HAVS is constituted by vibration-induced white finger (VWF), and typically cold conditions trigger it [3]. To evaluate VWF objectively, the cold excitation test has been utilized extensively [4]. In recent years, controversy has raged about the diagnostic ability of the cold excitation test in patients with VWF [5]. However, the use of electromyography for the diagnosis of HAVS has numerous limitations.

With the increasing prevalence of hand–arm vibration syndrome (HAVS), early identification of individuals at risk is essential for reducing long-term health consequences. However, current diagnostic approaches primarily rely on overt clinical symptoms and late-stage manifestations, particularly vascular signs such as VWF. As a result, the identification of biological changes that occur prior to meeting clinical diagnostic criteria remains challenging.

Against this backdrop, the aim of this study is to explore potential biomarkers associated with vibration exposure in workers, with a particular focus on individuals who do not yet meet the clinical diagnostic criteria for VWF or HAVS. In this context, the term “early” does not refer to disease onset or temporal progression, but rather to biomarker abnormalities observed in vibration-exposed workers in the absence of clinically diagnosed VWF or HAVS.

Specifically, abnormalities in these biomarkers, such as changes in expression levels, may reflect subclinical neurovascular alterations associated with vibration exposure. Our findings indicate that certain VWF-related markers already exhibit significant differences in some exposed workers without a formal VWF diagnosis, even though these alterations are not sufficient to satisfy current clinical diagnostic thresholds.

Through this approach, we aim to identify biomarkers that may serve as potential screening candidates for vibration-exposed populations, pending further validation in longitudinal studies.

The early symptoms of HAVS are not obvious. Oxidative stress (OS) indicators may be abnormal in the early stage of HAVS [1,6]. By detecting OS indicators, potential lesions can be detected before clinical symptoms appear, thereby enabling an early diagnosis. HAVS is closely related to OS [7]. Vibration exposure can lead to excessive production of ROS in the body, which exceeds the removal capacity of the antioxidant system. This phenomenon leads to OS and then damages vascular endothelial cells and neurons [8]. Dynamic monitoring of changes in indicators enables the OS of the disease at different stages to be understood, which provides a basis for targeted treatment plans [9,10]. The early biomarkers of HAVS can provide a reference for the early diagnosis and biomarker research of other occupational diseases [1].

HAVS involves peripheral nerve dysfunction, microvascular impairment, and musculoskeletal injury [11]. Sensory and motor nerve conduction velocities are widely used functional indicators for evaluating peripheral nerve damage in HAVS [12]. However, electrophysiological measurements mainly reflect functional outcomes and provide limited insight into the underlying biological mechanisms. From a complementary perspective, neurological and muscle-related biomarkers can offer additional mechanistic information relevant to HAVS.S100β, a calcium-binding protein primarily expressed by glial cells, is released following glial activation or injury and is considered a sensitive marker of peripheral nerve damage [13]. Vibration exposure has also been shown to alter the immune microenvironment. IL-10, an important anti-inflammatory cytokine, plays a key role in regulating neuroinflammation, and its secretion may be affected by vibration-induced immune imbalance [14,15].

Muscular involvement is another important component of HAVS. CK is a well-established biomarker of muscle injury and increased muscle membrane permeability, and elevated CK levels have been reported in vibration-related muscle damage, indirectly reflecting disease severity [16,17]. In addition, GDF-15, a stress-responsive cytokine, is upregulated under conditions of hypoxia and metabolic stress and has been associated with muscle injury and repair processes [18,19].

Together, the combined evaluation of nerve conduction parameters and multiple biological markers allows a more comprehensive assessment of nerve dysfunction, immune response, and muscle injury in HAVS, contributing to a better understanding of its underlying mechanisms.

We investigated the potential of indicators to serve as early biomarkers for HAVS diagnosis. We wished to offer references for the early diagnosis and biomarker research of HAVS. We measured the expression of six OS-associated indices in blood (malondialdehyde (MDA), superoxide dismutase (SOD), glutathione (GSH), glutathione peroxidase (GSH-PX), ROS, glutathione disulfide (GSSG)) and four neurological indices (S100β, IL-10, CK, GDF-15). Then, we integrated the relevant outcomes of a questionnaire on occupational disease. Subsequently, the relationship with VWF was analyzed.

## 2. Methods and Materials

### 2.1. Epidemiological Survey of Factory Workers

The judgment sampling method was employed to conduct research on the indices related to OS from a factory in Zhongshan City (Guangdong Province, China). Participants were partitioned into three groups in a 1:1:1 proportion. Each group was composed of 50 individuals. The group with VWF was composed of workers who had been exposed to vibration and had been diagnosed with VWF. The non-VWF group was made up of workers who had been exposed to vibration yet did not experience VWF. The control group comprised workers who were not exposed to vibration. All participants were male individuals of Han Chinese ethnicity.

VWF was identified using a standardized epidemiological case definition. Case classification required the presence of recurrent, well-demarcated episodic finger blanching triggered by hand–arm vibration, together with photographic documentation of blanching episodes. Symptom data were obtained using a structured questionnaire with predefined diagnostic criteria, and eligibility was restricted to workers with documented occupational exposure to hand–arm vibration. To enhance diagnostic reliability, photographic records and symptom profiles were independently evaluated by trained investigators. Participants with primary Raynaud’s phenomenon or other known peripheral vascular diseases were excluded. In this study, case classification was based on a standardized epidemiological definition applied at the time of assessment; severity staging systems (e.g., the Stockholm Workshop Scale) and objective vascular function tests were not used, and individuals classified as non-VWF were those who did not meet the predefined case criteria during the evaluation period. The exclusion criteria were: (1) individuals with primary Raynaud’s syndrome; (2) patients with a history of acute or chronic diseases; (3) drug use in the previous 2 weeks. The same method and procedures were applied to measure neurological indices. Forty individuals were in each group. The present study was exploratory in nature and based on a fixed cohort of vibration-exposed workers, which precluded prospective determination of sample size. In addition, owing to sample availability, the oxidative stress and neurological biomarker analyses were conducted in partially overlapping subsets of participants, resulting in different sample sizes across biomarker panels. Therefore, no formal a priori sample size or power calculation was performed, and the sample size was determined by feasibility. All participants included in each analysis met identical inclusion and exclusion criteria.

We designed an occupational epidemiological questionnaire based on relevant literature. We invited study participants to complete the questionnaire. The questionnaire covered six main aspects: (i) personal details; (ii) life traits; (iii) occupation-related aspects; (iv) hand-related symptoms (white knuckles, numbness, finger pain, cold hands); (v) medical history; (vi) health status and medication use within the previous 2 weeks.

### 2.2. Collection and Processing of Blood Samples

Participants had to sign an informed consent form. After an overnight fast, a nurse extracted 4 mL of blood from an elbow vein. Samples were placed in heparin-anticoagulated tubes, shaken gently for 30 s, and stored at 4 °C. Then, samples underwent centrifugation (3000× *g*, 10 min, 4 °C). Plasma was separated and stored in 500-μL tubes at −80 °C. For experiments, samples were thawed naturally at room temperature. Venous blood sampling protocols were executed in strict accordance with the procedures detailed in the publication authored by Kanshou Zhou [20].

### 2.3. Enzyme-Linked Immunosorbent Assay (ELISA)

Enzyme-linked immunosorbent assay (ELISA) kits supplied by MEIMIAN (Yancheng, China) were employed to determine the plasma expression levels of ten target molecules: MDA (MM-2037H1), GSH (MM-0458H1), SOD (MM-0390H1), GSH-Px (MM-60305H1), ROS (MM-1893H1), GSSG (MM-14753H1), CK (MM-0863H1), GDF-15 (MM-1643H1), IL-10 (MM-0066H1), and S100β (MM-13258H1) across the three study groups. All experimental operations were conducted in strict accordance with the manufacturer’s recommended protocols.

First, the required number of pre-coated microplate strips was taken out from the foil packaging after equilibration to ambient temperature for 20 min. Unused strips were preserved in a hermetically sealed bag at 4 °C for subsequent use. Standard wells were established, and 50 μL of standard solutions with gradient concentrations were added to their corresponding wells. For sample wells, 10 μL of each sample was pipetted followed by 40 μL of sample diluent, while blank wells were left unoccupied. Next, 100 μL of horseradish peroxidase (HRP)-conjugated detection antibody was added to all wells except the blank ones. The microplate was sealed with a plate sealer and incubated at 37 °C for 60 min.

After incubation, the liquid in each well was discarded, and the plate was patted dry on absorbent paper. Each well was then filled with wash buffer, allowed to stand for 1 min, and the liquid was discarded before patting dry again. This washing cycle was repeated five times, with the option of using an automated plate washer for efficiency. Subsequently, 50 μL of Substrate A and 50 μL of Substrate B were added to each well, followed by incubation at 37 °C for 15 min under light-protected conditions. A total of 50 μL of stop solution was then added to each well, and the optical density (OD) at 450 nm was measured within 15 min.

OD values of the standard solutions were recorded, and a regression curve was generated by plotting standard concentrations on the *x*-axis and their corresponding OD values on the *y*-axis. The regression curve was required to meet a precision criterion of R-squared (R^2^) ≥ 0.99 to qualify as the standard curve. This validated standard curve was further utilized to compute the expression concentrations of each target molecule in the tested samples [20].

### 2.4. Statistical Analyses

Statistical analyses were performed using SPSS version 26.0 (IBM, Armonk, NY, USA) and SAS version 9.4 (SAS Institute Inc., Cary, NC, USA). Categorical variables were analyzed using chi-square tests, and continuous variables were compared using one-way analysis of variance (ANOVA) with Bonferroni correction for pairwise comparisons.

Collinearity was assessed prior to regression analyses. Principal component analysis (PCA) was applied to oxidative stress indicators with high collinearity, whereas indicators without collinearity were directly included in regression models. Univariate logistic regression analysis was performed to evaluate the associations of oxidative stress indicators with VWF, while multivariate logistic regression analysis was conducted to assess the associations of neurological indicators with VWF, adjusting for potential confounding factors including age, duration of service, and BMI. ROC curve analysis was used to assess diagnostic performance.

Continuous variables are presented as mean ± standard deviation. Graphs were generated using Prism version 10.0 (GraphPad Software, La Jolla, CA, USA). A two-sided *p* value < 0.05 was considered statistically significant; given the hypothesis-driven nature of the analyses, no formal adjustment for multiple testing was applied.

## 3. Results

### 3.1. Descriptive Analyses of Epidemiology Data

#### 3.1.1. OS-Related Indicators

Each group consisted of 50 participants. The control group had an average age of 41.28 ± 8.45 years, and the average duration of service was 10.26 ± 7.55 years. The average age of the non-VWF group was 40.12 ± 7.21 years, and the average duration of service was 11.70 ± 7.92 years. The average age of the VWF group was 40.02 ± 6.28 years, and the average duration of service was 12.79 ± 7.40 years.

There were no significant differences among the three groups in terms of age (*p* = 0.638), duration of service (*p* = 0.254), body mass index (BMI; *p* = 0.299), prevalence of tobacco smoking (*p* = 0.923), or prevalence of alcohol consumption (*p* = 0.923). The prevalence of hand numbness, finger pain, and hand chills was markedly higher in the VWF group compared with that in the other two groups (*p* < 0.0001 for each factor) (Table 1).

#### 3.1.2. Neurological Indicators

Each group comprised 40 participants. The average age in the control group was 41.83 ± 7.15 years, and the average duration of service was 10.88 ± 8.06 years. The average age of the non-VWF group was 39.75 ± 6.81 years, and the average duration of service was 12.79 ± 8.39 years. The average age of the VWF group was 39.80 ± 5.98 years, and the average duration of service was 13.33 ± 7.50 years.

Among the three groups, significant differences were not observed with respect to age (*p* = 0.337), duration of service (*p* = 0.357), BMI (*p* = 0.422), prevalence of tobacco smoking (*p* = 0.587), or prevalence of alcohol consumption (*p* = 0.967). The prevalence of hand numbness, finger pain, and hand chills was significantly higher in the VWF group than that in the other two groups (*p* < 0.0001 for all) (Table 1).

### 3.2. Expression of Indicators

**Figure 1:**
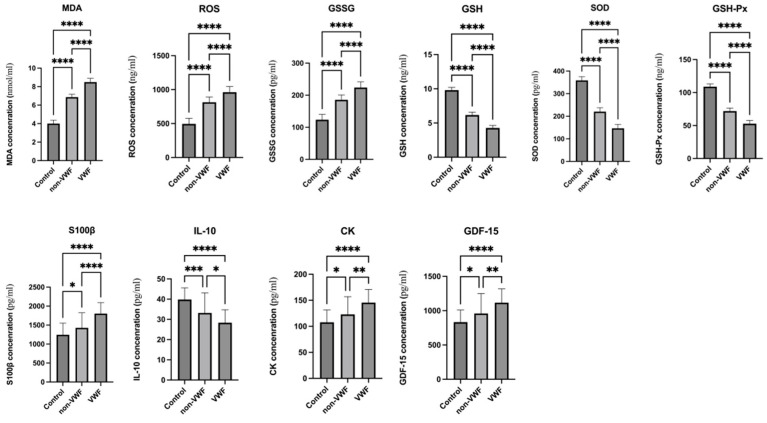
Expression of six indicators in the plasma of three groups of participants. Data are the mean ± standard deviation. * *p* < 0.05 ** *p* < 0.01 *** *p* < 0.001 **** *p* < 0.0001.

Compared with the control group (*p* < 0.0001) and non-VWF group (*p* < 0.0001), the expression of MDA, ROS, and GSSG in the VWF group was increased, and their expression in the non-VWF group was significantly higher than that in the control group (*p* < 0.0001). The expression of SOD, GSH, and GSH-Px in the VWF group was significantly lower than that in the control group (*p* < 0.0001) and non-VWF group (*p* < 0.0001), and their expression in the non-VWF group was significantly lower than that in the control group (*p* < 0.0001) (Figure 1).

The expression of S100β, CK, and GDF-15 was increased in the VWF group compared with that in the control group (*p* < 0.0001) and non-VWF group (*p* < 0.05), and their expression in the non-VWF group was significantly higher than that in the control group (*p* < 0.05). IL-10 expression in the VWF group was markedly lower than that in the control group (*p* < 0.0001) and non-VWF group (*p* < 0.05), and IL-10 expression in the non-VWF group was significantly lower than that in the control group (*p* < 0.001) (Figure 1).

### 3.3. Logistic Regression Analysis of Factors Affecting HAVS

#### 3.3.1. Collinearity Analysis of Oxidative Stress and Neurological Biomarkers

Before regression modeling, collinearity among candidate biomarkers was assessed. VWF among vibration-exposed workers was treated as the dependent variable, with age, duration of service, and BMI included as covariates.

Collinearity analyses were conducted separately for oxidative stress (OS) indicators (Table 2) and neurological biomarkers (Table 3). A high degree of multicollinearity was observed among the six OS indicators, whereas no substantial collinearity was detected among the four neurological biomarkers.

#### 3.3.2. Principal Component Analysis of Oxidative Stress Indicators

Given the pronounced multicollinearity among oxidative stress (OS) indicators, principal component analysis (PCA) was performed to derive uncorrelated composite variables. PCA yielded a single dominant component (PC1) with an eigenvalue of 5.65, explaining 94.2% of the total variance among the six OS indicators.

This PCA-derived OS component was subsequently entered into logistic regression analysis. After adjustment for age, duration of service, and BMI, the OS component was significantly associated with the prevalence of VWF, indicating an overall contribution of oxidative stress to HAVS (Table 4).

#### 3.3.3. Logistic Regression Analysis of Individual Biomarkers

To further explore associations between individual biomarkers and VWF, logistic regression analyses were conducted according to their collinearity characteristics.

Due to the strong intercorrelations among OS indicators, each OS biomarker was entered separately into logistic regression models. All biomarkers were standardized prior to analysis to facilitate comparability of effect estimates, with odds ratios expressed per one standard deviation increase (Table 5).

In contrast, as no significant collinearity was detected among neurological biomarkers, these indicators were simultaneously included in a multivariable logistic regression model. Neurological biomarkers were likewise standardized, and all regression analyses were adjusted for age, duration of service, and BMI (Table 6).

### 3.4. Analyses of ROC Curves

#### 3.4.1. VWF

**Figure 2:**
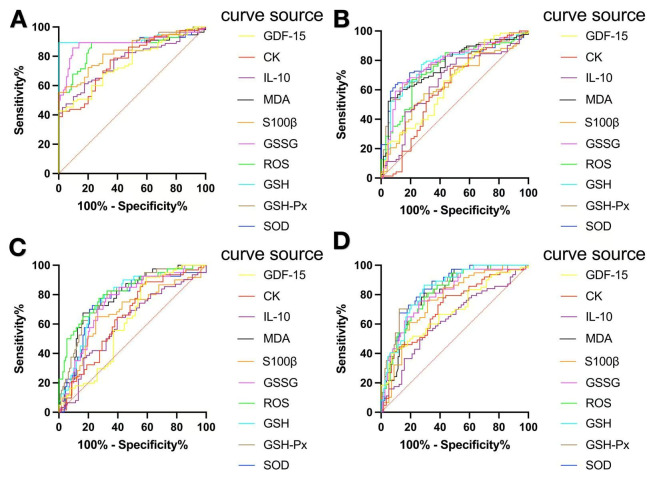
ROC curve of six indicators of HAVS-related symptoms. ROC curves for (**A**) VWF, (**B**) hand numbness, (**C**) hand pain, and (**D**) cold hands. The ROC curve closest to the upper-left corner implies increased sensitivity for this biomarker to diagnose HAVS and a reduced risk of false-positive results.

The characteristic curve of participants was analyzed to evaluate the sensitivity of indicators to VWF. Of the six OS-related indicators tested, the area under the ROC curve (AUC) of each index was >0.70 (Table 7 and Figure 2A). The cutoff value corresponding to the maximum Youden Index for SOD was 192.1 pg/mL (Table 7).

The AUC of each individual neurological indicator was >0.8. The threshold value corresponding to the AUC of S100β was 1375 pg/mL (Table 7).

#### 3.4.2. Numbness, Pain, and Cold Feeling in Hands

Typically, HAVS development is accompanied by alterations in VWF, as well as numbness and pain in the hand, along with the onset of cold sensations.

The ROC curve of hand numbness was analyzed. In tests of indicators related to OS, the AUC of each index was 0.70–0.80 (Table 8 and Figure 2B). SOD has the highest AUC (0.787; 95%CI = 0.714–0.861; *p* < 0.001), with a diagnostic threshold of 203.1 pg/mL (Table 8). When testing neurological indicators, the AUC of each indicator was 0.50–0.60 (Table 8 and Figure 2B). Among these indicators, IL-10 exhibited the highest AUC (0.652; 95%CI = 0.550–0.754; *p* < 0.01). The diagnostic cutoff for IL-10 was 30.66 pg/mL (Table 8).

In tests of indicators related to OS, the ROC curve for hand-prick pain revealed the AUC of each indicator to be 0.70–0.80 (Table 9) (Figure 2C). ROS had the highest AUC (0.815; 95%CI = 0.735–0.894; *p* < 0.001) with a diagnostic threshold of 858.2 ng/mL (Table 9). With regard to neurological measurements, the ROC curve revealed that the AUC of hand-prick pain was 0.60–0.70 (Table 9 and Figure 2C). Among these, S100β had the highest AUC (0.671; 95%CI = 0.570–0.771; *p* < 0.01). The diagnostic threshold for S100β was 1560 pg/mL (Table 9).

We analyzed the ROC curve for cold hands. In tests of indicators related to OS, the AUC for each indicator was 0.80–0.90 (Table 10 and Figure 2D). SOD had the highest AUC (0.845; 95%CI = 0.780–0.909; *p* < 0.001) with a diagnostic threshold of 160.8 pg/mL (Table 10). When conducting testing of neurological indicators, the AUC for each single indicator was 0.60–0.70 (Table 10 and Figure 2D). The AUC for S100β was 0.758 (95%CI = 0.669–0.846; *p* < 0.0001). The diagnostic threshold for S100β was 1560 pg/mL (Table 10).

## 4. Discussion

HAVS is difficult to cure, which seriously affects the health and quality of life of sufferers [21]. Even in the warm and humid southern cities of China, the incidence of HAVS among workers in industries such as polishing has shown an upward trend [22]. A “gold standard” diagnostic test for HAVS is lacking. Electromyography has drawbacks in terms of HAVS diagnosis. Therefore, seeking and validating indicators with high diagnostic validity and incorporating them as supplementary tools into a diagnostic system for HAVS is a rational approach.

### 4.1. Changes in the Expression Levels of Oxidative Stress-Related Indicators

#### 4.1.1. Multivariate Integration of Oxidative Stress Indicators (PCA)

In single-biomarker analyses, each oxidative stress indicator was significantly associated with vibration-induced white finger. When considered simultaneously in multivariable models, however, these markers exhibited pronounced collinearity, indicating that they do not act as independent predictors.

Principal component analysis revealed a dominant pattern in which markers of oxidative damage (MDA, ROS, and GSSG) loaded in opposition to antioxidant-related indicators (SOD, GSH-Px, and GSH). This configuration indicates a structured pattern of covariation among oxidative stress indicators, suggesting that individual biomarkers represent interrelated components of a common redox process rather than independent biological effects.

This structural relationship provides a statistical rationale for the integrated interpretation adopted below, in which oxidative stress in HAVS is discussed across three interconnected functional domains: oxidative burden, antioxidant capacity, and redox balance. It should be noted that PCA does not identify causal mechanisms but rather summarizes the dominant structure of intercorrelations among biomarkers, thereby facilitating a biologically coherent interpretation.

#### 4.1.2. Integrated Interpretation of Oxidative Stress Markers

Oxidative stress represents a dynamic process rather than a static biochemical state, involving continuous interaction between oxidant generation, antioxidant defense, and intracellular redox regulation [23]. In chronic exposure settings such as hand–arm vibration, individual oxidative stress markers frequently exhibit parallel changes and strong intercorrelations, reflecting their participation in the same biological cascade. Interpreting these markers independently may therefore fragment a fundamentally integrated process. To capture the biological coherence of oxidative stress progression in HAVS, the present findings are discussed within three interconnected functional domains—oxidative burden, antioxidant capacity, and redox balance—which together reflect different but mechanistically linked stages of redox dysregulation.

##### Oxidative Burden: Sustained Oxidant Generation and Propagation of Injury

Long-term vibration exposure imposes repetitive mechanical stress on vascular endothelium, peripheral nerves, and skeletal muscle [24,25], leading to persistent activation of oxidant-generating pathways. Mitochondrial dysfunction, characterized by impaired electron transport and increased electron leakage, represents a major source of excessive ROS under such conditions [26]. In parallel, activation of NADPH oxidases in vascular and neural tissues further amplifies ROS production [27]. This dual contribution results in sustained oxidative pressure rather than transient redox signaling.

ROS overproduction initiates lipid peroxidation by attacking polyunsaturated fatty acids within cellular and mitochondrial membranes [28]. MDA, as a stable end product of this process, therefore, reflects the cumulative and propagative nature of oxidant-induced membrane injury [29]. MDA has been used as a biomarker to measure oxidative stress in various biological samples such as blood [30]. In the present study, progressive increases in ROS and MDA from controls to non-VWF workers and further to the VWF group indicate that oxidative injury begins early during vibration exposure and accumulates over time. However, the elevation of these markers in non-VWF workers suggests that oxidant burden alone does not directly determine clinical disease but instead creates a permissive environment that enhances tissue vulnerability.

##### Antioxidant Capacity: Imbalance Between Oxidant Load and Enzymatic Defense

The biological impact of sustained oxidant generation is critically modulated by endogenous antioxidant systems [31]. SOD and GSH-Px form a coordinated enzymatic cascade that detoxifies superoxide radicals and hydrogen peroxide, thereby limiting the propagation of oxidative damage [10]. Under chronic vibration exposure, persistent ROS generation increases the demand placed on these enzymes, progressively exceeding their neutralizing capacity.

The observed decline in SOD and GSH-Px activities across exposure groups is consistent with antioxidant exhaustion driven by prolonged oxidative challenge. Reduced SOD activity permits superoxide accumulation, which not only directly damages cellular components but also reacts with nitric oxide, impairing endothelial function [32]. Simultaneously, diminished GSH-Px activity allows hydrogen peroxide and lipid hydroperoxides to accumulate, further amplifying lipid peroxidation and mitochondrial dysfunction [33]. This reciprocal relationship establishes a loop in which oxidative burden accelerates antioxidant depletion, thereby further increasing ROS availability.

Importantly, the early reduction in these enzymes in non-VWF workers suggests that loss of antioxidant capacity represents a transitional stage between compensated oxidative stress and overt tissue dysfunction, rather than a terminal event associated only with advanced disease.

##### Redox Balance: Collapse of Glutathione-Mediated Homeostasis

Beyond enzymatic defenses, cellular resilience to oxidative stress ultimately depends on maintenance of intracellular redox balance, which is largely governed by the glutathione system [34]. Reduced GSH acts as a central redox buffer [35]. In its reduced form, GSH donates electrons to neutralize ROS, including H_2_O_2_ and lipid peroxides, being oxidized to GSSG in the process [36].

The observed GSSG accumulation, together with concomitant GSH depletion, indicates a sustained shift toward an oxidized intracellular environment rather than a transient redox fluctuation. This shift has important functional consequences. First, reduced GSH availability limits the effectiveness of GSH-Px, further impairing peroxide detoxification and reinforcing oxidative injury [10]. Second, GSSG facilitates reversible S-glutathionylation of protein cysteine residues, a redox-sensitive post-translational modification that regulates protein structure and modulates signal transduction pathways in diverse physiological processes [37]. Under conditions of persistent oxidative burden, mitochondrial dysfunction and altered cellular energy metabolism may further limit the capacity for glutathione recycling, thereby reinforcing and stabilizing this oxidized redox state [26].

The observed trend in glutathione-related markers across the three groups suggests that disruption of redox homeostasis is not merely a downstream consequence of tissue damage, but a sustained and early feature of HAVS-related oxidative stress that bridges biochemical alterations and functional impairment.

### 4.2. Changes in the Expression Levels of Neurological Indicators

Among the investigated markers, S100β showed a clear increase with disease severity, indicating sustained peripheral neural stress. Mechanical vibration is known to affect nerve endings and support glial cells, leading to altered membrane permeability and glial activation [38]. The gradual elevation of S100β across exposure groups, therefore, likely reflects ongoing neural strain and functional disturbance, rather than transient injury, aligning with the progressive sensory impairment characteristic of HAVS.

In contrast, IL-10 exhibited a declining trend with increasing disease severity, indicating impaired anti-inflammatory regulation. IL-10 is a central cytokine involved in limiting inflammatory responses and maintaining immune homeostasis [39]. Experimental studies of vibration exposure have consistently reported persistent oxidative stress accompanied by low-grade inflammatory activation [40]. In this inflammatory context, reduced IL-10 levels have been associated with an inability to effectively counterbalance ongoing pro-inflammatory signaling. Sustained inflammatory activity and oxidative stress have been implicated in both vascular dysfunction and peripheral nerve impairment in vibration-related disorders [41], suggesting that diminished IL-10–mediated regulation may be relevant to key pathological features of HAVS.

CK levels increased gradually from exposed workers to patients with VWF, suggesting a cumulative effect of repeated mechanical loading on muscle tissue. Previous occupational studies have reported higher serum CK in workers using vibrating tools, even in the absence of clinically apparent muscle injury. Similar CK elevations have also been observed after experimental vibration or mechanical stress and are generally interpreted as reflecting mild muscle strain and increased membrane permeability rather than overt muscle damage [42]. Although CK lacks disease specificity, its consistent association with exposure intensity and disease severity supports its relevance to muscle involvement in HAVS.

GDF-15 is widely regarded as a stress-responsive cytokine linked to mitochondrial dysfunction and cellular stress adaptation [43]. Its elevation in vibration-exposed workers, therefore, suggests an ongoing cellular stress response under chronic mechanical exposure. Rather than indicating established tissue damage, increased GDF-15 is more consistent with sustained metabolic and mitochondrial stress, reflecting the cellular response to prolonged vibration-related strain.

It should be acknowledged that the neurological and muscle-related biomarkers examined in this study are not specific to HAVS and may also be influenced by non-HAVS determinants, such as physical workload and metabolic status. However, limited disease specificity does not negate their relevance as early biological indicators. Rather, these markers reflect neural stress, muscle strain, and cellular stress responses that may emerge in vibration-exposed workers before the development of overt clinical manifestations.

Therefore, the translational value of these biomarkers lies not in their use as standalone diagnostic indicators, but in providing complementary biological information when interpreted alongside exposure history and clinical symptoms. Although the term “early biomarkers” is used in this study, it does not imply prediction of future HAVS or VWF onset. Rather, “early” refers to biomarker abnormalities observed in vibration-exposed workers who do not meet current clinical diagnostic criteria for VWF, reflecting potential subclinical neurovascular or oxidative alterations. These findings support a potential screening role, while longitudinal studies are required to establish predictive value.

### 4.3. ROC Curve Analysis

ROC curves are employed frequently to assess the performance of binary classification models [44]. The ROC curve is a widely utilized tool for appraising the diagnostic performance of biomarkers in relation to specific diseases. The AUC serves as an index to gauge the accuracy of diagnostic tests. The AUC spans from 0.5 to 1.0. A higher Youden Index indicates greater accuracy of the screening test [45].

Various OS-related indicators were employed as diagnostic factors, and comprehensive ROC analyses were carried out for four typical symptoms of HAVS: VWF along with pain, numbness, and coldness in the hand. Among OS-related indices, the AUCs of MDA, SOD, and GSH-Px were > 0.80. The AUCs of S100β, IL-10, CK, and GDF-15 were >0.70. These data suggest that these biochemical markers possess high diagnostic potential for VWF. The threshold values for each indicator are presented in Table 7, Table 8, Table 9 and Table 10.

The AUC derived from ROC analysis reflects the discriminatory ability of a biomarker at the population level, rather than its direct clinical diagnostic value in individual patients. A higher AUC indicates better separation between diseased and non-diseased groups, but it does not imply that the biomarker can be applied independently for clinical decision-making.

Accordingly, the cut-off values identified in this study should be regarded as statistically defined thresholds that optimize sensitivity and specificity under the study conditions rather than fixed diagnostic criteria. In clinical practice, the usefulness of any given threshold depends on the clinical context, including exposure history and symptom presentation. Therefore, the AUC and corresponding cut-off values reported here are intended to demonstrate the potential discriminatory performance of the biomarkers, while their clinical application requires integration with other diagnostic information [46]. As these cut-off values were derived and evaluated within the same dataset, some degree of optimism bias cannot be excluded, and the present findings should therefore be regarded as hypothesis-generating. Validation in independent cohorts will be required before any occupational screening thresholds can be proposed.

## 5. Conclusions

This study demonstrates that oxidative stress- and neurobiology-related biomarkers represent early biological indicators of hand–arm vibration syndrome. Alterations in oxidative stress markers (MDA, SOD, GSH, and GSH-Px) and neurological biomarkers (S100β, IL-10, CK, and GDF-15) were detectable in vibration-exposed workers before overt clinical manifestations of vibration-induced white finger, indicating subclinical biological disturbances associated with HAVS. Principal component analysis provides a coherent representation of early redox dysregulation. Supported by favorable ROC performance, these biomarkers may serve as complementary early indicators when interpreted alongside exposure history and clinical symptoms.

This study has several limitations that merit consideration. Restriction to male Han Chinese workers and the use of a judgmental (non-probability) sampling strategy may limit generalizability. In addition, the cross-sectional design precludes causal inference and assessment of temporal relationships. Accordingly, the findings should be regarded as exploratory, and validation in longitudinal studies and independent cohorts is required before clinical or occupational screening applications.

## Figures and Tables

**Table 1:** Comparison of data in three groups of workers.

	Group	Control	Non-VWF	VWF	*F*/*x*^2^	*p*
**Age, x ± s**	Oxidative stress	41.28 ± 8.45	40.12 ± 7.21	40.02 ± 6.28	0.45	0.638
	Neurological	41.83 ± 7.15	39.75 ± 6.81	39.80 ± 5.98	1.10	0.337
**Length of service, x ± s**	Oxidative stress	10.26 ± 7.55	11.70 ± 7.92	12.79 ± 7.40	1.38	0.254
	Neurological	10.88 ± 8.06	12.79 ± 8.39	13.33 ± 7.50	1.04	0.357
**BMI, x ± s**	Oxidative stress	23.91 ± 3.78	24.89 ± 3.23	24.00 ± 3.44	1.22	0.299
	Neurological	23.83 ± 3.83	24.78 ± 3.06	23.96 ± 3.60	0.87	0.422
**Smoking, *n* (%)**	Oxidative stress	25 (50.00)	27 (54.00)	26 (52.00)	0.160	0.923
	Neurological	23 (57.50)	19 (47.50)	19 (47.50)	1.067	0.587
**Drinking, ** ***n*** ** (%)**	Oxidative stress	25 (50.00)	27 (54.00)	26 (52.00)	0.160	0.923
	Neurological	20 (50.00)	21 (52.50)	21 (52.50)	0.067	0.967
***Numb, n (%)***	Oxidative stress	15 (30.00)	27 (54.00)	46 (92.00)	40.304	<0.0001
	Neurological	14 (35.00)	23 (57.50)	38 (95.00)	31.360	<0.0001
**Pain, ** ***n*** ** (%)**	Oxidative stress	3 (6.00)	10 (20.00)	26 (52.00)	28.898	<0.0001
	Neurological	3 (7.50)	8 (20.00)	21 (52.50)	22.074	<0.0001
**Cold, *n* (%)**	Oxidative stress	1 (2.00)	9 (18.00)	27 (54.00)	38.173	<0.0001
	Neurological	1 (2.50)	7 (17.50)	27 (67.50)	44.854	<0.0001

**Table 2:** Collinearity diagnostics of oxidative stress indicators.

Variable	*β*	SE	*t*	*p*	Tol	VIF
**MDA**	0.457	0.077	0.590	0.553	0.045	22.400
**ROS**	−0.001	0.000	−1.370	0.173	0.141	7.094
**GSH**	−0.005	0.070	−0.070	0.943	0.036	28.050
**GSH-Px**	−0.005	0.006	−0.830	0.407	0.442	22.606
**GSSG**	0.003	0.002	1.540	0.126	0.127	7.851
**SOD**	−0.001	0.002	−0.490	0.624	0.044	22.824

**Table 3:** Collinearity diagnostics of neurological biomarkers.

Variable	*β*	SE	*t*	*p*	Tol	VIF
**S100β**	−0.000	0.000	−4.030	0.000	0.693	1.443
**IL-10**	0.010	0.004	2.490	0.014	0.811	1.233
**CK**	−0.004	0.001	−3.400	0.001	0.794	1.260
**GDF-15**	−0.000	0.000	−3.470	0.001	0.817	1.225
**S100β**	−0.000	0.000	−4.030	0.000	0.693	1.443
**IL-10**	0.010	0.004	2.490	0.014	0.811	1.233

**Table 4:** Association between a PCA-derived oxidative stress component and VWF.

Factors	*β*	SE	Wald *χ*^2^	*p*	OR (95%CI)
**OS (PCA-derived component)**	−2.375	0.426	31.003	<0.001	0.093 (0.040–0.215)

**Table 5:** Univariate logistic regression analysis of oxidative stress indicators. (per one standard deviation increase).

Factors	*β*	SE	Wald *χ*^2^	*p*	OR (95%CI)
**MDA**	2.264	0.397	32.448	<0.001	9.621 (4.415–20.966)
**ROS**	1.713	0.307	31.231	<0.001	5.547 (3.042–10.117)
**GSH**	−2.333	0.420	30.887	<0.001	0.097 (0.043–0.221)
**GSH-Px**	−2.317	0.417	30.838	<0.001	0.099 (0.043–0.223)
**GSSG**	2.028	0.343	34.926	<0.001	7.595 (3.877–14.879)
**SOD**	−2.293	0.409	31.467	<0.001	0.101 (0.045–0.225)

Note: Odds ratios for continuous biomarkers are reported per one standard deviation increase.

**Table 6:** Multivariable logistic regression analysis of neurological indicators (per one standard deviation increase).

Factors	*β*	SE	Wald *χ*^2^	*p*	OR (95% CI)
**S100β**	−1.240	0.363	11.637	<0.001	0.289 (0.142–0.590)
**IL-10**	0.734	0.338	4.730	<0.05	2.084 (1.075–4.038)
**CK**	−0.955	0.349	7.478	<0.01	0.385 (0.194–0.763)
**GDF-15**	−0.969	0.349	16.070	<0.01	0.380 (0.198–0.729)

Note: Odds ratios for continuous biomarkers are reported per one standard deviation increase.

**Table 7:** AUC parameters and threshold values of indicators to diagnose VWF.

Index	AUC Parameters					Judgment Boundary Value				
	AUC	SE	*p*	95%CI	Cutoff Value	Sensitivity	Specificity	Youden Index	+LR	−LR
**MDA**	0.909	0.036	0.000	0.839–0.979	7.406	0.893	0.979	0.872	41.920	0.109
**ROS**	0.858	0.036	0.000	0.787–0.929	838.300	0.893	0.777	0.670	3.997	0.138
**GSH**	0.918	0.031	0.000	0.854–0.982	5.506	0.893	0.979	0.872	41.920	0.109
**GSH-Px**	0.923	0.030	0.000	0.864–0.983	65.300	0.893	0.979	0.872	41.920	0.109
**GSSG**	0.888	0.034	0.000	0.821–0.955	197.700	0.857	0.904	0.761	8.956	0.158
**SOD**	0.916	0.033	0.000	0.850–0.982	192.100	0.893	0.979	0.872	41.920	0.109
**S100β**	0.835	0.036	0.000	0.764–0.906	1375.000	0.592	0.950	0.542	11.842	0.429
**IL-10**	0.768	0.042	0.000	0.685–0.850	34.720	0.613	0.825	0.438	3.500	0.470
**CK**	0.770	0.043	0.000	0.685–0.855	139.200	0.775	0.650	0.425	2.214	0.346
**GDF-15**	0.753	0.044	0.000	0.666–0.840	840.100	0.453	0.950	0.403	9.066	0.575

**Table 8:** AUC parameters and threshold values of indicators to diagnose hand numbness.

Index	AUC Parameters					Judgment Boundary Value				
	AUC	SE	*p*	95%CI	Cutoff Value	Sensitivity	Specificity	Youden Index	+LR	−LR
**MDA**	0.769	0.039	0.000	0.693–0.846	7.406	0.546	0.936	0.481	8.457	0.486
**ROS**	0.746	0.041	0.000	0.666–0.826	836.900	0.6705	0.790	0.461	3.197	0.417
**GSH**	0.779	0.039	0.000	0.703–0.855	6.478	0.7727	0.726	0.499	2.818	0.313
**GSH-Px**	0.784	0.038	0.000	0.71–0.858	67.730	0.614	0.871	0.485	4.757	0.444
**GSSG**	0.777	0.039	0.000	0.7–0.854	191.300	0.6477	0.855	0.503	4.461	0.412
**SOD**	0.787	0.038	0.000	0.714–0.861	203.100	0.636	0.903	0.540	6.574	0.403
**S100β**	0.641	0.052	<0.05	0.540–0.742	1423.000	0.574	0.708	0.282	1.966	0.602
**IL-10**	0.652	0.052	<0.01	0.550–0.754	30.660	0.761	0.551	0.312	1.694	0.434
**CK**	0.616	0.055	<0.05	0.508–0.724	139.200	0.747	0.531	0.277	1.590	0.478
**GDF-15**	0.646	0.053	<0.01	0.542–0.749	1288.000	0.941	0.298	0.239	1.341	0.197

**Table 9:** AUC parameters and threshold values of indicators to diagnose hand pain.

Index	AUC Parameters					Judgment Boundary Value				
	AUC	SE	*p*	95%CI	Cutoff Value	Sensitivity	Specificity	Youden Index	+LR	−LR
**MDA**	0.787	0.040	0.000	0.709–0.865	3.635	0.675	0.836	0.511	4.126	0.389
**ROS**	0.815	0.041	0.000	0.735–0.894	858.200	0.775	0.727	0.502	2.842	0.309
**GSH**	0.780	0.040	0.000	0.701–0.859	5.992	0.825	0.673	0.498	2.521	0.260
**GSH-Px**	0.793	0.040	0.000	0.715–0.871	69.590	0.800	0.673	0.473	2.444	0.297
**GSSG**	0.748	0.043	0.000	0.664–0.833	191.300	0.775	0.682	0.457	2.436	0.330
**SOD**	0.753	0.046	0.000	0.663–0.843	193.400	0.725	0.773	0.498	3.190	0.356
**S100β**	0.671	0.051	<0.01	0.570–0.771	1560.000	0.650	0.750	0.400	2.600	0.467
**IL-10**	0.607	0.054	<0.05	0.502–0.712	35.040	0.645	0.604	0.249	1.628	0.588
**CK**	0.647	0.051	<0.01	0.547–0.747	106.600	0.855	0.448	0.303	1.549	0.324
**GDF-15**	0.618	0.054	<0.05	0.511–0.724	731.800	0.918	0.407	0.325	1.549	0.201

**Table 10:** AUC parameters and threshold values of indicators to diagnose hand cold.

Index	AUC Parameters					Judgment Boundary Value				
	AUC	SE	*p*	95%CI	Cutoff Value	Sensitivity	Specificity	Youden Index	+LR	−LR
**MDA**	0.804	0.037	0.000	0.731–0.877	7.676	0.730	0.797	0.526	3.586	0.339
**ROS**	0.810	0.037	0.000	0.737–0.883	846.000	0.811	0.681	0.492	2.545	0.278
**GSH**	0.833	0.035	0.000	0.765–0.901	5.900	0.865	0.708	0.573	2.962	0.191
**GSH-Px**	0.833	0.037	0.000	0.760–0.907	57.200	0.703	0.876	0.579	5.672	0.339
**GSSG**	0.800	0.041	0.000	0.719–0.881	197.700	0.757	0.743	0.500	2.949	0.327
**SOD**	0.845	0.033	0.000	0.780–0.909	160.800	0.676	0.876	0.552	5.454	0.370
**S100β**	0.758	0.045	0.000	0.669–0.846	1560.000	0.763	0.684	0.447	2.415	0.347
**IL-10**	0.626	0.051	<0.05	0.525–0.726	35.290	0.556	0.684	0.240	1.759	0.650
**CK**	0.708	0.047	0.000	0.615–0.801	137.200	0.778	0.579	0.357	1.847	0.384
**GDF-15**	0.691	0.049	<0.01	0.595–0.786	749.100	0.400	0.929	0.329	5.602	0.646

## Data Availability

The data supporting the findings of this study are available from the corresponding author upon request and have not been publicly disclosed due to privacy or ethical restrictions. The raw research data generated from this study have been included in the Supplementary Materials.

## Supplementary Materials

The following supporting information can be downloaded at: https://www.mdpi.com/article/10.3390/biom16020238/s1, Table S1: Control Group Questionnaire; Table S2: Data organization; Table S3: Vibration Group Questionnaire.
